# Glucagon-like peptide-2 protects the gastric mucosa *via* regulating blood flow and metabolites

**DOI:** 10.3389/fendo.2022.1036559

**Published:** 2022-12-16

**Authors:** Jing Zhang, Jing Ning, Xinyu Hao, Xiurui Han, Wei Fu, Yueqing Gong, Qiao Meng, Shigang Ding, Jing Zhang

**Affiliations:** Department of Gastroenterology, Peking University Third Hospital, Beijing, China

**Keywords:** GLP-2, gastroprotection, gastric mucosal blood flow, metabolomics, linoleic acid metabolism

## Abstract

**Introduction:**

Refractory peptic ulcers lead to perforation and hemorrhage, which are fatal. However, these remain a therapeutic challenge. Gastric mucosal blood flow is crucial in maintaining gastric mucosal health. It’s reported that Glucagon-like peptide-2 (GLP-2), a gastrointestinal hormone, stimulated intestinal blood flow. However, the direct role of GLP-2 in gastric mucosal blood flow and metabolites remain unclear. Here, we speculated that GLP-2 might protect the gastric mucosa by increasing gastric mucosal blood flow and regulating metabolites. This study was conducted to evaluate the role of GLP-2 in gastric mucosal lesions and its underlying mechanism.

**Methods:**

We analyzed endogenous GLP-2 during gastric mucosal injury in the serum. Rats were randomly divided into two groups, with 36 rats in each group as follows: (1) normal control group (NC1); (2) ethanol model group (EC1); rats in EC1 and NC1 groups were intragastrically administered ethanol (1 ml/200 g body weight) and distilled water (1 ml/200 g body weight). The serum was collected 10 min before intragastric administration and 15, 30, 60, 90, and 120 min after intragastric administration. Furthermore, additional male Sprague–Dawley rats were randomly divided into three groups, with six rats in each group as follows: (1) normal control group (NC); (2) ethanol model group (EC); (3) 10 μg/200 g body weight GLP-2 group (GLP-2). Rats in the NC and EC groups were intraperitoneally injected with saline. Those in the GLP-2 group were intraperitoneally injected with GLP-2. Thirty minutes later, rats in the EC and GLP-2 groups were intragastrically administered ethanol (1 ml/200 g body weight), and rats in the NC group were intragastrically administered distilled water (1 ml/200 g body weight). After the intragastric administration of ethanol for 1 h, the animals were anesthetized and gastric mucosal blood flow was measured. Serum were collected for ultra performance liquid chromatography/tandem mass spectrometry (UPLC-MS/MS) metabolomics.

**Results:**

There were no significant change in endogenous GLP-2 during gastric mucosal injury (P<0.05). Pretreatment with GLP-2 significantly reduced ethanol-induced gastric mucosal lesions by improving the gastric mucosal blood flow, as examined using a laser Doppler flow meter, Guth Scale, hematoxylin-eosin staining, and two-photon microscopy. UPLC-MS/MS analyses showed that GLP-2 also maintained a steady state of linoleic acid metabolism.

**Conclusions:**

Taken together, GLP-2 protects the gastric mucosa against ethanol-induced lesions by improving gastric mucosa blood flow and affecting linoleic acid metabolism.

## Introduction

Gastric ulcer is a kind of gastric mucosal disease. The prevalence is approximately 5–10% in the general population (1). The health of gastric mucosal depends on the balance between defense and injury-related factors. Drinking alcohol, *H. pylori* infection, and the widespread use of nonsteroidal anti-inflammatory drugs are injury-related factors (2, 3). The five defense factors are mucus and bicarbonate, epithelial cells, tight junction proteins, immunity, and gastric mucosal blood flow (4). Standard pharmacotherapy concentrates on the use of potent acid-suppressing proton-pump inhibitors, H_2_-receptor antagonists, and antibiotics to eliminate *Helicobacter pylori* (*H. pylori*) (5, 6). In most cases, ulcers can be healed under standard treatments. However, in some situations, conventional treatment is ineffective, which is called refractory peptic ulcer (7). Perforation and hemorrhage are both complications of gastric ulcer, which introduces marked risk. Gastric mucosal blood flow is of great importance in maintaining gastric mucosal health as it continuously supplies oxygen and nutrients and removes H^+^ and toxic agents diffusing into the mucosa. A reduction in gastric mucosal blood flow can lead to gastric ulcer (8). Therefore, increasing gastric mucosal blood flow might be a practical therapy for refractory peptic ulcers.

Glucagon-like peptide-2 (GLP-2), composed of 33 amino acids, is a gastrointestinal hormone belonging to the progucagon-derived peptide family. GLP-2 is secreted by intestinal L cells (9)and performs its functions *via* the glucagon-like peptide-2 receptor (GLP-2R), which is a G protein-coupled receptor. Previous studies have shown that GLP-2R is expressed on the membranes of gastrointestinal submucosal cells (10, 11). In neonatal pigs, GLP-2 infusion dose-dependently stimulates intestinal blood flow *via* eNOS/NO (10). However, the direct role of GLP-2 in gastric mucosal blood flow and its influence on metabolites remain unclear. Here, we speculated that GLP-2 might protect the gastric mucosa by increasing gastric mucosal blood flow and regulating metabolites.

Therefore, we established a rat model of ethanol-induced gastric mucosal lesions to explore the function of GLP-2 in the gastric mucosa and gastric mucosal blood flow. Metabolomics were used to reveal the underlying etiology and elucidate the function of GLP-2 in gastric lesions.

## Materials and methods

### Animals, chemicals, and antibodies

Male Sprague–Dawley rats weighing 250–300 g was selected from the Experimental Animal Center, Peking University Health Science Center [certification: SYXK (Jing) 2006-0025, LA2018321]. The animal experiments were approved by the Ethics Committee of the Peking University Health Science Center in accordance with the Regulations for the Administration of Affairs Concerning Experimental Animals (Beijing, China). Rats were housed at 4–6 per cage under constant environmental conditions (20–24°, 12 h light-dark cycle). The animals were fasted for 24 h before the experiments, with free access to tap water up until 1 h before testing.

Rats were randomly divided into three groups, with six rats in each group as follows (1): normal control group (NC) (2); ethanol model group (EC) (3); 10 μg/200 g body weight GLP-2 group (GLP-2). Rats in the NC and EC groups were intraperitoneally injected with saline. Those in the GLP-2 group were intraperitoneally injected with GLP-2. Thirty minutes later, rats in the EC and GLP-2 groups were intragastrically administered ethanol (1 ml/200 g body weight), and rats in the NC group were intragastrically administered distilled water (1 ml/200 g body weight). After the intragastric administration of ethanol for 1 h, the animals were anesthetized and examined using a laser Doppler flow meter, Guth Scale, hematoxylin-eosin staining, and two-photon microscopy. The Ultra performance liquid chromatography/tandem mass spectrometry (UPLC-MS/MS) analyses were applied to detect the metabolites in the serum

Rat GLP-2 (His-Ala-Asp-Gly-Ser-Phe-Ser-Asp-Glu-Met-Asn-Thr-Ile-Leu-Asp-Asn-Leu-Ala-Thr-Arg-Asp-Phe-Ile-Asn-Trp-LeuIle-Gln-Thr-Lys-Ile-Thr-Asp) was synthesized by the Chinese peptide company (Hangzhou, China). An antibody against GLP-2R (GLP2R-201AP) was from FabGennix (San Francisco, USA).

### Gastric mucosal blood flow measurement

Each animal in EC, NC and GLP-2 group was anesthetized *via* the intraperitoneal injection of pentobarbital (60 mg/kg body weight). Their body temperature was maintained at 36° using a homeothermic blanket. A laparotomy was performed through a midline epigastric incision. The stomach was exposed and brought to the abdominal surface using gentle traction. The gastric mucosa was washed three times with saline and then bathed with 1.5 ml of saline. The entire gastric mucosal surface was measured using a laser Doppler flow meter (MoorFLPI-2, Moor Instruments Ltd, Devon, UK) connected to a computer with the matching software moorVMS-PC v3.1 (Moor) to process data. The animals were euthanized *via* pentobarbital.

### *In vivo* paracellular permeability assay

To establish an *in vivo* vessel permeability detection system, 40 kDa fluorescein isothiocyanate-dextran (Sigma-Aldrich, Burlington, MA, USA) was used. The powder was dissolved in saline away from light. Then, 20 mg/200 g body weight fluorescein isothiocyanate-dextran was injected into the caudal vein of each animal in EC, NC and GLP-2 group. The stomach was exposed, as described above. The gastric mucosa was observed under a 2-photon microscope (Leica TCS SP8, Wetzlar, Germany). Images were taken every 15 min for areas that included the blood vessels to evaluate paracellular permeability.

### Enzyme-linked immunosorbent assay

To exclude the effects of endogenous GLP-2 during gastric mucosal injury, we measured the GLP-2 concentration in the serum. Rats were randomly divided into two groups, with 36 rats in each group as follows (1): normal control group 1 (NC1) (2); ethanol model group 1 (EC1); rats in the EC1 group were intragastrically administered ethanol (1 ml/200 g body weight), and rats in the NC1 group were intragastrically administered distilled water (1 ml/200 g body weight). The serum was taken 10 min before the intragastric administration of ethanol or distilled water. Serum was also taken to evaluate GLP-2 concentrations at 15, 30, 60, 90, and 120 min after the ethanol-mediated induction of gastric mucosal lesions. Serum samples were obtained *via* centrifugation at 16000 × *g* for 10 min. GLP-2 (EK-028-14, Phoenix Pharmaceuticals, Burlingame, CA, USA) was measured following the manufacturer’s instructions.

### Measurements of gastric mucosal damage

After gastric mucosal blood flow measurement, stomachs of each animal in EC, NC and GLP-2 group were removed and scored for gastric mucosal damage following the Guth Scale Standard (**Table 1**) (12).

**Table 1:** Guth scale standard.

Score	gastric mucosa damage length(L)
0	no lesion
1	L<1 mm
2	1 mm ≤ L< 2 mm
3	2 mm ≤ L< 3 mm
4	3 mm ≤ L< 4 mm
5	L ≥ 4 mm
The total score summed over the whole stomach.	

### Pathological examination of gastric mucosa

Strip-shaped tissue specimens from the gastric antrum of animals in EC, NC and GLP-2 groups to the corpus were quickly cut off on ice. The specimens were fixed in neutral buffered formaldehyde for 24 h at room temperature and embedded in paraffin. Sections of 5 µm were cut and stained with hematoxylin-eosin for histopathological evaluation.

### UPLC-MS/MS analyses

Serum samples from each animal in EC, NC and GLP-2 group were subjected to UPLC-MS/MS analysis (Realbio, Shanghai, China). Six serum samples were obtained from each group. After the addition of 400 μl of extract solution (acetonitrile:methanol = 1:1, containing isotopically labeled internal standard mixture) to 100 μl serum, the samples were vortexed for 30 s, sonicated for 10 min in an ice-water bath, and incubated for 1 h at −40° to precipitate proteins. The sample was centrifuged at 13800 × *g* for 15 min at 4°. The quality control (QC) sample was prepared by mixing an equal aliquot of the supernatant from all samples. UPLC-MS/MS analyses were performed using an ultra high pressure liquid chromatography system (Vanquish, Thermo Fisher Scientific, Waltham, MA, USA) with a ultra pressure liquid chromatography BEH Amide column (2.1 mm × 100 mm, 1.7 μm) coupled with an Orbitrap Exploris 120 mass spectrometer (Orbitrap MS, Thermo Fisher). The injection volume was 2 μl. The raw data can be accessed from https://www.ebi.ac.uk/metabolights/MTBLS6123.

The relative standard deviation values of the retention time and peak area of the QC samples were positively and negatively analyzed. Principal component analysis (PCA) and orthogonal partial least squares discriminant analysis (OPLS-DA) methods were used to classify groups suspected of showing differences in metabolism. We used the Kyoto Encyclopedia of Genes and Genomes (KEGG, http://www.genome.jp/kegg/) database to clarify the functions and biological relevance of many metabolites (13) and to perform enrichment analysis for each metabolic pathway based on the differential metabolites. The standard *P-*value was set to 0.05 to indicate a statistical difference. Using PCA and OPLS-DA, we compared the metabolites of linoleic acid metabolism among the NC, EC, and GLP-2 groups.

### Statistical analysis

The entire database was statistically analyzed using GraphPad Prism software (version 8.0). A Student’s *t-*test or ANOVA was used to compare differences between or among groups, and statistical significance was set at *P<* 0.05.

## Results

### No significant change in endogenous GLP-2 during gastric mucosal lesions

**Figure 1:**
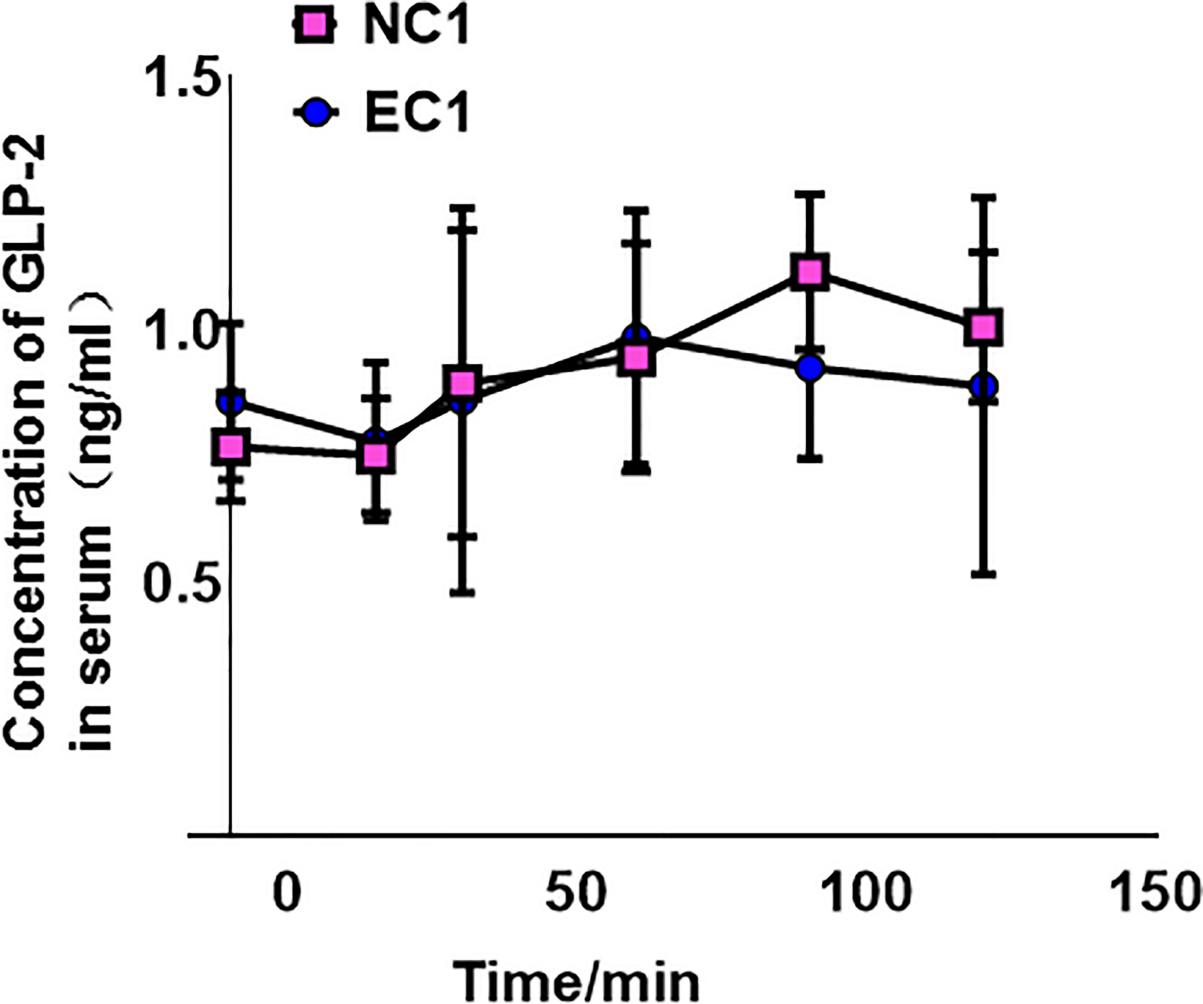
Concentration of glucagon-like peptide-2 (GLP-2) in normal control 1 (NC1) and ethanol model 1 (EC1) groups, presented as means ± SD.

A comparison of GLP-2 measurements before and at the specified time points after GLP-2 injection revealed no significant change in GLP-2 after gastric injury (**Figure 1**).

### GLP-2 reduces ethanol-induced gastric mucosal lesions

**Figure 2:**
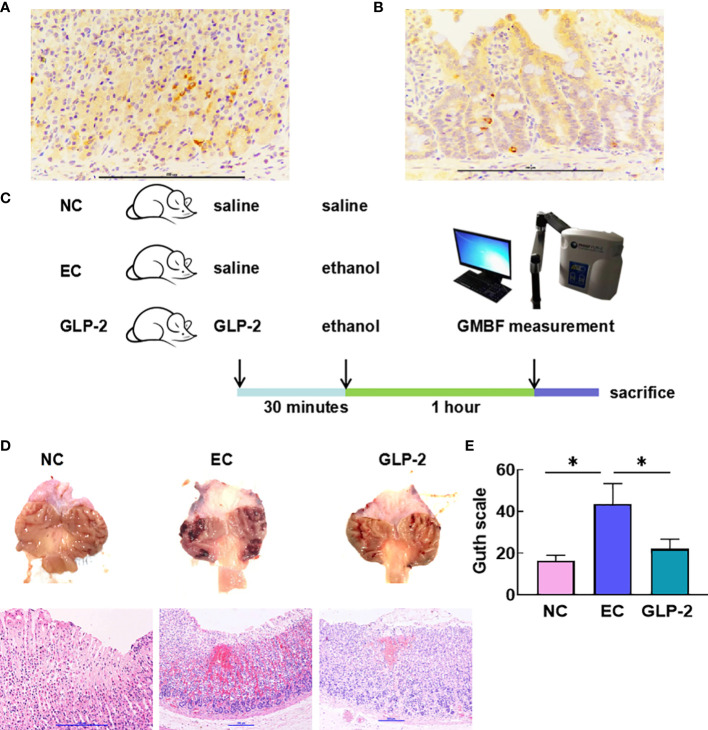
Effect of GLP-2 on the macroscopic and microscopic appearance of the ethanol-treated gastric mucosa among normal control (NC), ethanol model (EC) and glucagon-like peptide-2 (GLP-2) groups. **(A, B)** Distributions of glucagon-like peptide-2 receptor (GLP-2R) in the stomach **(A)** and colon tissue as a positive control **(B)**. **(C)** Schematic of the animal experiments. **(D)** Macroscopic appearance (upper), haematoxylin-eosin staining (lower). **(E)** The gastric injury index according to the Guth scale. Data are presented as the mean ± SD. *P < 0.05.

Many studies have reported the distribution of GLP-2R in the stomach, but these lack protein-level results. We determined the expression of GLP-2R in the stomach using immunohistochemistry (**Figure 2A**) and used colon tissue as a positive control (**Figure 2B**). Based on the features of GLP-2R distribution, we speculated that GLP-2R was mainly distributed on the neuroendocrine cells of the stomach.

To validate our experimental approach (**Figure 2C**), we explored the function of GLP-2 in gastric tissue. Histomorphology revealed that ethanol administration caused severe gastric mucosal lesions, such as diffuse edema and linear hemorrhagic zones. The GLP-2-pretreated group had fewer lesions with reduced lengths and numbers of hemorrhagic stripes. We also observed destruction of the surface epithelium, submucosal edema, hemorrhagic injury, and mucosal distortion *via* microscopic observation in the EC group. Nevertheless, the GLP-2 group showed a reduction in hemorrhagic areas (**Figure 2D**). The injury level in the GLP-2 group was significantly lower than that in the EC group (**Figure 2E**).

### GLB-2 improves gastric mucosal blood flow by reducing capillary permeability

**Figure 3:**
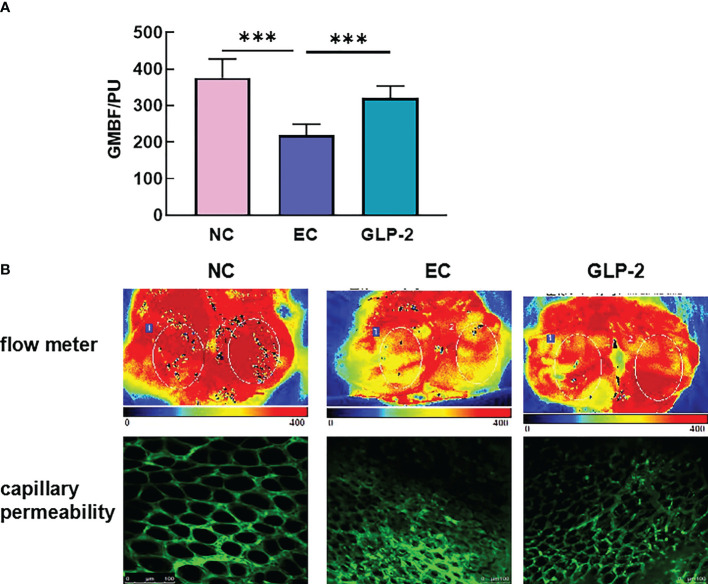
Effect of GLP-2 on the gastric mucosal blood flow in the ethanol-induced gastric mucosa among normal control (NC), ethanol model (EC) and glucagon-like peptide-2 (GLP-2) groups. **(A)** Changes in gastric mucosal blood flow measured using a laser Doppler flow meter. **(B)** gastric mucosal blood flow heat plot based on a laser Doppler flow meter (upper), vascular morphology, and capillary permeability using a 2-photon microscope (lower). ***P < 0.001.

As a vital gastric mucosal barrier, gastric mucosal blood flow maintains a healthy gastric mucosa. Ethanol significantly decreased gastric mucosal blood flow in the EC group compared with that in the NC group, and GLP-2 increased gastric mucosal blood flow after ethanol-induced gastric mucosal lesions (**Figure 3A**). The heat map of the blood flow volume revealed that ethanol induced the reduction in gastric mucosal blood flow mainly on the gastric antrum (**Figure 3B**, upper). Vascular permeability and morphology are crucial factors that determine gastric mucosal blood flow. The two-photon microscope was applied to detect the leakage of fluorescein isothiocyanate-dextran,which reflected the microvascular permeability. Ethanol induced an increase in microvascular permeability, which was reduced by pretreatment with GLP-2 (**Figure 3B**).

### Metabolic profiling of plasma samples in ethanol-induced gastric mucosal lesions

**Figure 4:**
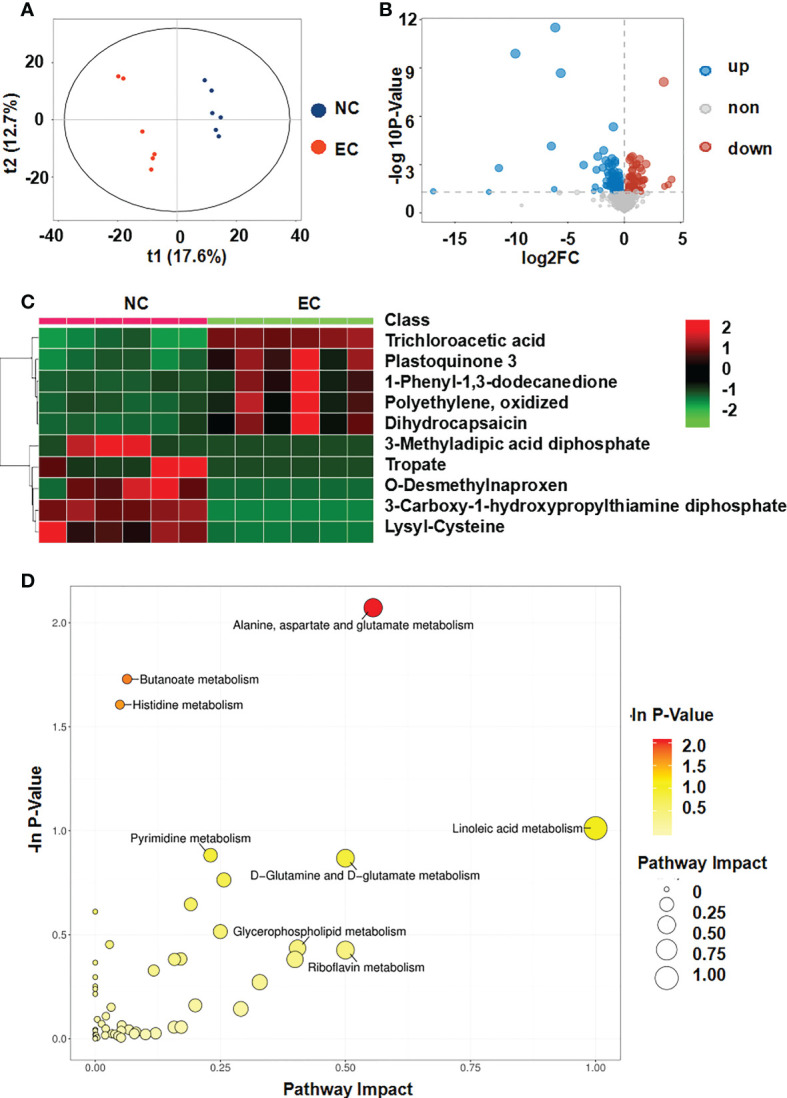
Metabolomic analysis of the serum of rats after administering ethanol. **(A)** Score plots of the orthogonal projections to latent structure-discriminant analysis (OPLS-DA) model between NC and EC groups. **(B)** Volcano plot of differential metabolites. **(C)** Community heatmap of the differential metabolites. **(D)** Bubble plots of pathway analysis between NC and EC groups.

The OPLS-DA score plot demonstrated that the EC group differed from NC normal controls, suggesting that metabolic fluctuation occurred in the EC group (**Figure 4A**). Metabolite data were acquired in the positive and negative modes. Metabolites with variable importance in the projection (VIP) values ≥ 1, a fold-change ≥ 2 or ≤0.5, and a *P*-value ≤ 0.05, were selected as differential metabolites. The NC rats had 41 upregulated and 25 downregulated metabolites relative to levels in the EC rats, which were considered potential biomarkers for gastric mucosal lesions (**Table 2**). To characterize the plasma metabolite profile, we generated a volcano plot of the differential metabolites (**Figure 4B**) and a heat map of the top 10 differential metabolites (**Figure 4C**). The KEGG enrichment analysis showed that in rats with gastric lesions, metabolism was abnormal, mainly with respect to the metabolism of alanine, aspartate, glutamate, butanoate, and histidine (**Figure 4D**).

**Table 2:** 41 upregulated and 25 downregulated metabolites in normal control (NC)vs. ethanol model (EC) rats.

Metabolites	p_value	FC(NC/EC)	VIP_score
Tropate	0.00	0.00	1.53
3-Methyladipic acid	0.00	0.00	1.41
O-Desmethylnaproxen	0.00	0.00	2.05
3-Carboxy-1-hydroxypropylthiamine diphosphate	0.00	0.00	2.53
Lysyl-Cysteine	0.00	0.01	2.28
3-(2-Furanyl)-2-propenal	0.00	0.01	1.55
Mannose 6-phosphate	0.00	0.01	2.53
dCMP	0.00	0.02	2.50
trans-Ferulic acid	0.00	0.08	2.13
Gravelliferone	0.00	0.15	1.41
8-Hydroxypinoresinol 4-glucoside	0.00	0.17	1.67
Hydroxyphenylacetylglycine	0.00	0.18	2.00
Aminoadipic acid	0.00	0.20	2.23
Estriol	0.00	0.23	1.45
4-Hydroxy-3-methylbenzoic acid	0.00	0.27	2.03
N-Carbamoyl-2-amino-2-(4-hydroxyphenyl)acetic acid	0.00	0.28	2.23
PE(P-18:0/24:0)	0.00	0.32	2.14
Thioguanine	0.00	0.32	1.72
(9Z,11R,12S,13S,15Z)-12,13-Epoxy-11-hydroxy-9,15-octadecadienoic acid	0.00	0.33	1.80
12S-HHT	0.00	0.34	1.70
Hydroxyprolyl-Hydroxyproline	0.00	0.36	1.92
Xanthosine	0.00	0.38	1.85
alpha-Tocopherol	0.00	0.38	2.14
2-Hydroxybutyric acid	0.00	0.39	2.07
Hydroxyoctanoic acid	0.00	0.40	1.63
Succinic acid semialdehyde	0.00	0.42	1.82
PC(18:3(6Z,9Z,12Z)/18:3(6Z,9Z,12Z))	0.01	0.42	1.79
N4-Acetylsulfamethoxazole	0.01	0.45	1.75
[3-[1-Formyl-2-(2-furanyl)ethenyl]]-2-(2-furanyl)-5-(2-furanylmethylene)-4,5-dihydro-a-methyl-4-oxo-1H-pyrrole-1-acetic acid, 9CI	0.01	0.46	1.84
3,4-Dihydroxy-9-methoxypterocarpan	0.01	0.46	1.87
2,4,6-Trimethoxyphenyl acetate	0.01	0.47	1.84
Erythrulose	0.01	0.47	2.22
Catechin	0.01	0.47	2.04
N-Acetyl-L-methionine	0.01	0.47	2.07
PC(20:5(5Z,8Z,11Z,14Z,17Z)/15:0)	0.01	0.48	1.52
Isosakuranin	0.01	0.49	1.50
Xylitol	0.01	0.49	1.97
PC(16:1(9Z)/14:1(9Z))	0.01	0.49	1.61
Avenalumin II	0.01	0.49	1.81
Indoxyl sulfate	0.01	0.50	1.66
1H-Indole-2,3-dione	0.01	0.50	1.88
3-Hydroxyquinine	0.01	2.04	2.07
17-Hydroxyprogesterone	0.01	2.05	1.76
L-Anserine	0.01	2.05	1.72
LysoSM(d18:1)	0.02	2.17	2.10
Prolylhydroxyproline	0.02	2.17	1.91
Hexadecanedioic acid mono-L-carnitine ester	0.02	2.20	1.60
Taurochenodeoxycholate	0.02	2.22	1.80
Small bacteriocin	0.02	2.23	1.46
4-Hydroxyestrone	0.02	2.38	1.90
4-Hydroxycitrulline	0.02	2.48	1.75
Glycohyocholic acid	0.02	2.54	1.57
3-Methylglutarylcarnitine	0.03	2.55	2.16
Cholesterol sulfate	0.03	2.60	1.57
Histidinal	0.03	2.61	2.06
(Â±)-Anisoxide	0.03	2.79	1.54
N1,N10-Diferuloylspermidine	0.03	2.90	1.98
Lucidenic acid E2	0.03	2.96	1.82
Harmine	0.03	3.05	1.58
4,8 Dimethylnonanoyl carnitine	0.04	3.20	1.80
Medicagenic acid 28-O-[b-D-xylosyl-(1->4)-a-L-rhamnosyl-(1->2)-a-L-arabinosyl] ester	0.04	3.42	1.88
Plastoquinone 3	0.04	3.79	2.16
Trichloroacetic acid	0.05	11.22	2.50
1-Phenyl-1,3-dodecanedione	0.05	11.84	1.72
Polyethylene, oxidized	0.05	14.90	1.78
Dihydrocapsaicin	0.05	18.23	1.91

### Effects of GLP-2 on the plasma metabolic profiles

**Figure 5:**
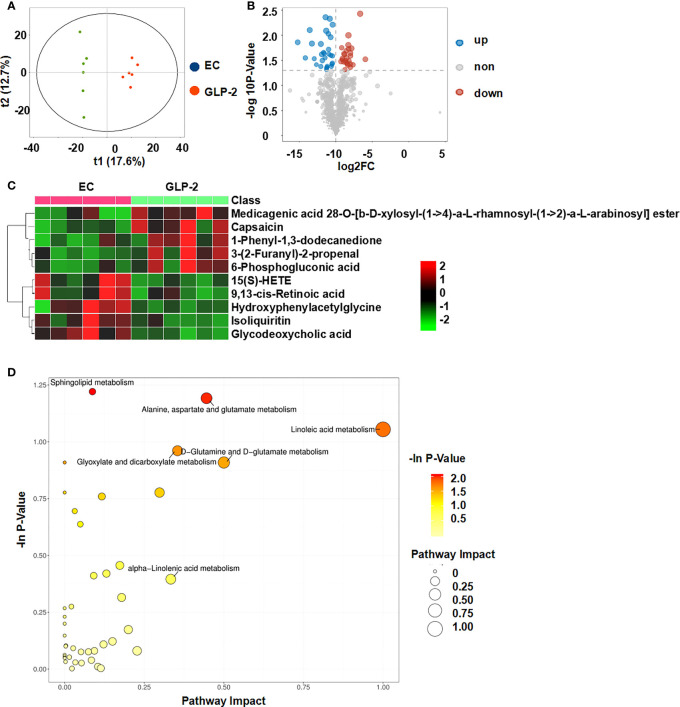
Metabolomic analysis of the serum of rats after GLP-2 treatment. **(A)** Score plots of the orthogonal projections to latent structure-discriminant analysis (OPLS-DA) model for the EC and GLP-2 groups. **(B)** Volcano plot of differential metabolites. **(C)** Community heatmap of the differential metabolites. **(D)** Bubble plots of pathway analysis between the EC and GLP-2 group.

Because GLP-2 is a hormone that can affect metabolism, we explored the mechanism that GLP-2 reduces ethanol-induced gastric mucosal lesions and improves gastric mucosal blood flow by UPLC-MS/MS analysis. We compared the differential metabolites in the serum between the EC and GLP-2 groups. Considering the void supervision of the PCA model, OPLS-DA was employed. Using this approach, distinct clusters were obtained between the EC and GLP-2 groups (**Figure 5A**). Differential metabolites were selected based on the following parameters: VIP value ≥ 1, fold-change ≥ 2 or ≤ 0.5, and *P*-value ≤ 0.05. There were 28 upregulated and 26 downregulated metabolites in the serum of EC-groups rats compared to GLP-2 group rats (**Table 3**). Volcano plots were used to display the metabolite profiles (**Figure 5B**), and a heat map of the top 10 differential metabolites was generated to show the differential metabolites (**Figure 5C**). KEGG pathway enrichment analysis identified matched pathways based on *P-*values ≤ 0.05. The results suggested that linoleic acid metabolism was the main influencing pathway in the serum of the GLP-2 treatment group (**Figure 5D**).

**Table 3:** 28 upregulated and 26 downregulated metabolites in ethanol model (EC) vs. glucagon-like peptide-2 (GLP-2) rats.

Metabolites	p_value	FC(EC/GLP-2)	VIP_score
9,13-cis-Retinoic acid	0.03	3.08	2.19
Glycodeoxycholic acid	0.00	2.52	2.44
Hydroxyphenylacetylglycine	0.04	1.93	2.02
15(S)-HETE	0.02	1.82	2.33
Isoliquiritin	0.03	1.75	2.10
PC(22:6(4Z,7Z,10Z,13Z,16Z,19Z)/20:3(5Z,8Z,11Z))	0.02	1.74	2.24
LysoPE(15:0/0:0)	0.04	1.73	2.11
hesperetin 3’-O-sulfate	0.04	1.72	2.23
8-HETE	0.03	1.65	2.18
Benzoic acid	0.02	1.63	2.16
13-OxoODE	0.01	1.62	2.47
7-Methylinosine	0.02	1.61	2.38
12-HEPE	0.04	1.57	2.18
(Â±)-2-(1-Methylpropyl)-4,6-dinitrophenol	0.04	1.55	2.02
Hypoxanthine	0.03	1.50	2.26
PC(22:4(7Z,10Z,13Z,16Z)/16:0)	0.02	1.49	2.00
12-Hydroxydodecanoic acid	0.03	1.48	2.12
Isolinderanolide	0.03	1.46	1.95
Erythrulose	0.05	1.42	1.97
10E,12Z-Octadecadienoic acid	0.02	1.40	2.16
Linoleic acid	0.02	1.37	2.23
N-Acetylserine	0.03	1.36	2.11
Petroselinic acid	0.03	1.29	2.19
SM(d18:1/20:0)	0.03	1.29	1.87
Pentadecanoic acid	0.02	1.25	2.01
L-Aspartyl-4-phosphate	0.03	1.23	2.27
Fluticasone propionate	0.01	0.89	2.27
Aconine	0.04	0.88	1.98
LysoPC(18:1(11Z))	0.03	0.88	1.94
PS(18:1(9Z)/15:0)	0.04	0.86	1.92
Homocitrulline	0.04	0.85	1.89
1-(2,3-Dihydro-1H-pyrrolizin-5-yl)-1,4-pentanedione	0.03	0.83	1.83
PC(P-16:0/20:3(5Z,8Z,11Z))	0.01	0.82	2.24
1-O-Hexadecyl-2-O-dihomogammalinolenoylglycero-3-phosphocholine	0.00	0.80	2.37
Ecgonine methyl ester	0.02	0.77	1.89
PE(P-18:1(11Z)/22:5(4Z,7Z,10Z,13Z,16Z))	0.02	0.76	2.03
1-(beta-D-Ribofuranosyl)-1,4-dihydronicotinamide	0.01	0.76	2.10
Musanolone D	0.04	0.72	1.79
Propionylcarnitine	0.04	0.72	1.85
N-Acetylhistamine	0.04	0.71	1.87
Ginkgotoxin	0.05	0.71	1.81
Montecristin	0.01	0.70	2.45
Calabaxanthone	0.00	0.68	2.35
Cysteinyl-Histidine	0.03	0.67	1.87
Methylmalonic acid	0.02	0.65	1.96
Quercetin 8-C-(2’’-rhamnosylglucoside)	0.02	0.63	1.96
Nb-Lignoceroyltryptamine	0.03	0.60	2.06
N-Acetyldehydroanonaine	0.02	0.59	2.07
3-Hydroxyquinine	0.01	0.57	2.30
PI(20:2(11Z,14Z)/16:0)	0.04	0.57	1.91
4-Hydroxycitrulline	0.04	0.48	1.73
1-Phenyl-1,3-dodecanedione	0.03	0.44	1.85
Medicagenic acid 28-O-[b-D-xylosyl-(1->4)-a-L-rhamnosyl-(1->2)-a-L-arabinosyl] ester	0.01	0.40	2.32
Capsaicin	0.01	0.37	2.29
6-Phosphogluconic acid	0.03	0.32	2.00
3-(2-Furanyl)-2-propenal	0.01	0.24	2.19

### Linoleic acid metabolism is involved in the gastric protective effect of GLP-2

**Figure 6:**
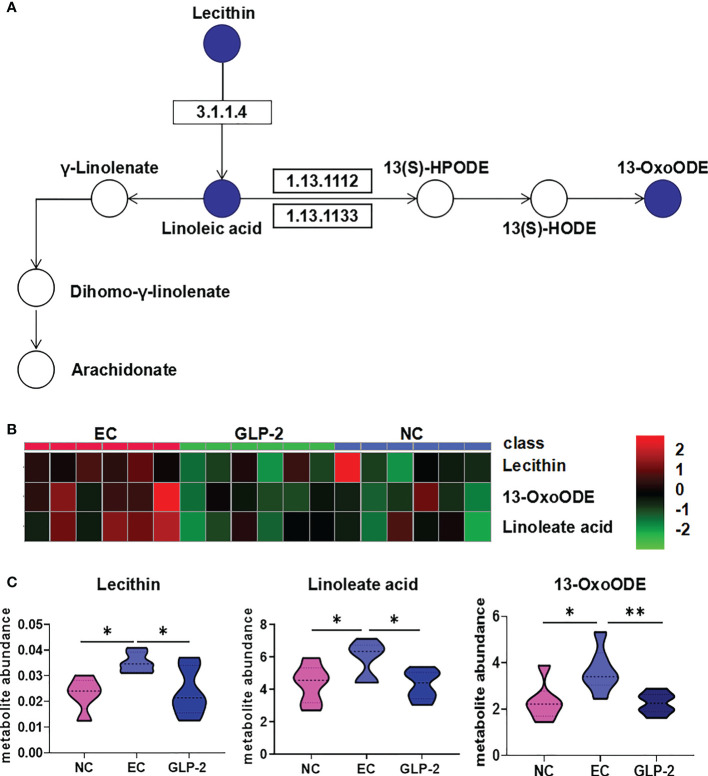
Change in linoleic acid metabolism pathway. **(A)** Differential metabolites of the linoleic acid metabolism pathway in the NC, EC, and GLP-2 groups. The blue circle indicates the differential metabolites. **(B, C)** the relative abundances of the main metabolites in the linoleic acid metabolism pathway in the NC, EC, and GLP-2 groups based on metabolomic analyses are displayed in the heat map **(B)** and violin plot **(C)**. *P < 0.05, **P < 0.01.

Linoleic acid metabolism was the only significantly different pathway (*P* ≤ 0.05) between the EC and GLP-2 groups (**Figure 6A**). Lecithin, linoleate, and 13-OxoODE were elevated in ethanol-induced gastric mucosal lesions. GLP-2 pretreatment inhibited this elevation in ethanol-induced lecithin, linoleate, and 13-OxoODE (**Figures 6B, C**).

## Discussion

Gastric mucosal blood flow is of great importance in maintaining the health of the gastric mucosa as it supplies oxygen and ${\text{HCO}}_{3}^{-}$ to the mucosa and removes H^+^ and toxic agents diffusing from the lumen into the mucosa (8). The reduction in blood flow resulting in the accumulation of H^+^, which is the main reason for gastric mucosal lesions. Increased oxygen supply mediated by gastric mucosal blood flow is crucial for ulcer healing (14, 15). Ethanol-induced gastric mucosal lesions were associated with gastric microvascular congestion and stasis and a decrease in mucosal blood flow. Thereby, ethanol-induced gastric mucosal lesions in rats are an ideal and widely used model to study gastric mucosal blood flow (16–18). In our study, gastric mucosal blood flow was reduced in rats with ethanol-induced gastric mucosal lesions, which is consistent with the results from previous study (19). Systemic and local metabolic factors regulate gastric mucosal blood flow, such as prostaglandin, calcitonin gene-related peptides (CGRP), and other endogenous chemical mediators (20, 21). It has been reported that the intracerebroventricular injection of GLP-2, a gastrointestinal hormone, prevents the decrease in gastric mucosal blood flow induced by CGRP receptor activation (13, 15). However, there are limited data on the peripheral effects of GLP-2 on gastric mucosal blood flow. Our study firstly revealed that GLP-2 increased gastric mucosal blood flow by reduction of vascular permeability.

Many biological activities occur at the level of metabolites, such as cell signaling, energy transfer, and intercellular communication, which are regulated by these compounds. Metabolomics helps us understand the overall metabolic image of gastric mucosal lesions (22). Gastric mucosal injury leads to perturbations in lipid, amino acid, and energy metabolism (23–26). Our findings provide novel insights into the mechanism of gastric mucosa injury. And the identification of potential biomarkers may be further investigated for diagnosis and therapy. Alanine is an important participant and regulator of glucose metabolism and can augment mucosal blood flow during gut ischemia/reperfusion (27). Glutamate has protective effects against gastric ischemia-reperfusion injury in rats (28). Butanoate metabolism is related to the maintenance of epithelial cells and regulates intestinal immunity (29). Studies are lacking in the role of butanoate metabolism on gastric mucosal blood flow, which requires further investigation. Histamine is a crucial component of histidine metabolism. Gastric microcirculation and histamine concentrations have been reported to decline markedly when acute gastric injury occurs, indicating that histamine might play an important role in gastric microcirculation (30).

Linoleic acid and oxidized linoleic acid metabolites have been reported to increase during ischemia-reperfusion (26, 31). Many studies have discovered the connection between linoleic acid metabolism and inflammation (32). Oxidized lipid product 13-oxoODE have been mechanistically connected to pathological conditions ranging from cardiovascular disease to chronic pain (33, 34). We speculate that GLP-2 alleviates gastric lesions by reducing oxidized lipid products, which provides a new anti-inflammatory mechanism of GLP-2. Besides, the oxidized lipid products can lead to endothelial cell dysfunction which is responsible for the abnormal vascular permeability (35). But there are no direct reports about the linoleic acid metabolism and gastric mucosal blood flow. We speculate that GLP-2 may decrease vascular permeability by reducing oxidized lipid product and protecting endothelial cell. Because lecithin is the upstream metabolite in the linoleic acid metabolism pathway, we presume the main target of GLP-2 is lecithin, which need a further study. Our study firstly revealed the overall metabolic image of GLP-2.

This study focused on the connection between gastric mucosal blood flow and metabolic changes in a gastric injury model, offering a deeper understanding of the pathology underlying gastric mucosal ischemia, and contributes to the development of new therapeutic approaches for refractory peptic ulcer. However, there are some limitations in our study. Since this study carried out on the animal model, it cannot be stated with certainty whether it applies to clinical situations. Serum from gastric ulcer patients should be collected to determine the increase of oxidized linoleic acid metabolites. Second, a series of *in vivo* and *in vitro* experiments are needed to determine the function of related metabolic pathways on gastric mucosal blood flow. We will intervene the related metabolic pathways in animal model to figure out metabolic effects on the gastric mucosal blood flow and vascular permeability in the future.

## Conclusion

GLP-2 could be capable of protecting the gastric mucosa against ethanol-induced lesions by regulating gastric mucosal blood flow and linoleic acid metabolic pathway. Lecithin maybe the main target of GLP-2, which need a further investigation.

## Data availability statement

The original contributions presented in the study are publicly available. This data can be found here: https://doi.org/10.6084/m9.figshare.21723434.v1.

## Ethics statement

The animal study was reviewed and approved by Ethic Committee of Peking University Health Science Center.

## Author contributions

JZ (1st Author) and YG: data curation and writing—original draft preparation. XYH, WF, XRH, and QM: methodology. JN and SD: conceptualization. JZ (9th Author): supervision and writing—reviewing and editing. All authors contributed to the article and approved the submitted version.

## Funding

This study was supported by the National Natural Science Foundation of China (Grant No. 11702004).

## Conflict of interest

The authors declare that the research was conducted in the absence of any commercial or financial relationships that could be construed as a potential conflict of interest.

## Publisher’s note

All claims expressed in this article are solely those of the authors and do not necessarily represent those of their affiliated organizations, or those of the publisher, the editors and the reviewers. Any product that may be evaluated in this article, or claim that may be made by its manufacturer, is not guaranteed or endorsed by the publisher.

## Abbreviations

EC, ethanol model group; GLP-2, glucagon-like peptide-2; GLP-2R, glucagon-like peptide-2 receptor; KEGG, Kyoto Encyclopedia of Genes and Genomes; NC, normal control group; OPLS-DA, orthogonal partial least squares discriminant analysis; PCA, Principal component analysis; QC, quality control; UPLC-MS/MS, ultra performance liquid chromatography/tandem mass spectrometry; VIP, Variable importance in the projection.
